# Surgical and Functional Outcomes of the Results of Conventional Two-Screw Proximal Femoral Nail (PFN) Versus Helical-Blade Anti-rotation Proximal Femoral Nail (PFNA2)

**DOI:** 10.7759/cureus.43698

**Published:** 2023-08-18

**Authors:** Manish R Shah, Manisha M Shah, Isha M Shah, Karma R Shah

**Affiliations:** 1 Orthopedics, Sumandeep Vidyapeeth (Deemed to be University), Vadodara, IND; 2 Pathology, Medical College Baroda, Vadodara, IND; 3 Orthopedics, GMERS (Gujarat Medical Education and Research Society) Medical College, Gotri, Vadodara, IND

**Keywords:** intertrochanteric fracture, trochanter fracture, pfna2, pfn, proximal femoral nail

## Abstract

Introduction

An intertrochanteric (IT) femur fracture is an extra-capsular fracture between greater and lesser trochanters. Unstable IT fractures are those where there is poor contact between fracture fragments (especially medial and posterior cortices), comminution, and fracture pattern, such that the weight-bearing forces tend to displace the fracture further or a reverse oblique type. Proximal femoral nailing (PFN) is one of the modalities for proximal femoral fractures. A newer modality for proximal femoral fracture is PFNA2, i.e., PFN anti-rotation, which makes use of a helical blade for a better compaction of bone. Both nail designs (PFN and PFNA2) are available in short and long sizes (so a total of four variants). Only a few studies have compared the treatment of IT femur fracture concerning fracture geometry, design, and length using either of the two nail types. In our study, we assessed the surgical and functional outcomes of PFN and PFNA2.

Materials and methods

This prospective observational study was carried out on 30 patients who had sustained IT fractures of the femur. All cases of IT femur fractures more than 18 years of age, closed injuries, and the patients who consented to participate in this program were included in the study. All open injuries, the patients who refused to participate in this program, patients who have associated injuries, patients with a subtrochanteric femur fracture, and patients with less than six months of follow-up were excluded. The patients were randomized into two types of implant groups. All patients were operated with a standard protocol. The study was conducted for 18 months from February 2021 to August 2022. The results were analyzed (of all four variants) by comparing patient demographics, implant size, implant type, locking methods, union time, and other parameters.

Results

Most of the patients were operated on with a 10 mm nail diameter (17/30 patients), 380 mm length (long-nail group) (five/11 patients), and 250 mm length (short-nail group). With the use of PFNA2, the overall duration of hospital stay was less. The overall operative time (incision to wound closure) with the use of the short PFNA2 was lesser than that with the use of other designs due to the use of the zig for distal screws. The use of a distal dynamic locking screw in a majority of the patients can get better compression at the fracture site once the patient starts weight bearing and decrease the chances of the Z-effect, reverse Z-effect, screw back-out, and screw cut-out. The union time was nearly the same in the majority of the patients, with an early union seen with the use of PFNA2 nails. The overall modified Harris hip score (HHS) at the final follow-up was nearly the same with slightly better results with the use of PFNA2.

Conclusions

PFNA2 is the implant of choice in elderly patients with osteoporotic bone. It has less operative time, which is required in such patients with medical comorbidities; hence, it has marginal superiority over PFN. Short-nail design results in less operative time and less blood loss.

## Introduction

An intertrochanteric (IT) femur fracture is an extra-capsular fracture between greater and lesser trochanters. It accounts for nearly 50% of all proximal femur fractures and is a major cause of disability in the elderly. The incidence of IT femur fracture is gender and race dependent varying demographically [1]. Several epidemiological studies in Asian countries suggest increasing incidence and general life expectancy during the last few decades [2]. In 1997, Gulberg et al. suggested that the worldwide incidence of hip fractures would double to 2.6 million by 2025 and 4.5 million by 2050 [3]. The incidence of proximal femoral fractures among females is two to three times higher than the incidence of such fractures in males [4]. Hagino et al. reported a lifetime risk of hip fractures for individuals at 50 years of age of 5.6% for men and 20% for women [5]. Cummings et al. noted that neither age-related osteoporosis nor the increasing incidence of falls with age sufficiently explains the exponential increase the hip fracture with aging [6].

Unstable IT fractures are those where there is poor contact between fracture fragments (especially medial and posterior cortices), comminution, and fracture pattern, such that the weight-bearing forces tend to displace the fracture further or a reverse oblique type [7]. The treatment has evolved and changed over a period of time, from conservative to operative, from open reductions and fixations to closed reduction and fixation and newer minimally invasive techniques. Among the treatment modalities, there are two types of devices, i.e., intramedullary and extramedullary. Intramedullary devices, such as the gamma nail (GN) and proximal femoral nail (PFN), have the same theoretical advantage over the dynamic hip screw (DHS) because they do not depend on the screw fixation of a plate to the lateral cortex, which can be a worrying part in osteoporotic bone. For PFN, Fogagnolo et al. found that the intraoperative technical or mechanical complication rate is as high as 23.4% [8].

PFN is one of the modalities for proximal femoral fractures. A newer modality for proximal femoral fracture is PFNA2, i.e., PFN anti-rotation, which makes use of a helical blade for a better compaction of the bone.

In PFN, two screws are used for fixation; the larger femoral neck screw carries most of the load. The smaller hip screw will provide rotational stability. In PFNA2, there is a single proximal blade that compacts the cancellous bone. Both nail designs (PFN and PFNA2) are available in short and long sizes. Only a few studies have compared the treatment of IT femur fracture with both designs. None of them have compared the results with respect to fracture geometry, design, and length using either of the two nail types. As per our literature search, no similar studies are available. In our study, we assessed the surgical and functional outcomes of PFN and PFNA2 (both with short and long varieties, so a total of four variants).

## Materials and methods

After getting clearance from the Ethical Committee of Sumandeep Vidyapeeth, the prospective observational study was carried out on 30 patients admitted to Dhiraj Hospital, Piparia, Vadodara, Gujarat, India, who had sustained IT fracture of the femur after getting the written informed consent.

We included all the patients who met the inclusion and exclusion criteria for the study during February 2021 to August 2022. The sample size was calculated by the following formula:

S=Z^2 ^X P X (1-P)/M^2^,

where S = sample size, Z = Z-score (1.96), P = population proportion (assumed as 50% or 0.5), and M = margin of error.

All the data were entered in Microsoft Excel (Microsoft Corporation, United States). All the quantitative data were presented in mean and appropriate tables, and graphical methods were used for the presentation of the data. For qualitative data, numbers and percentages were used, and appropriate graphs were used for the presentation of the data.

All cases of IT femur fractures more than 18 years of age, closed injuries, and patients who consented to participate in this program were included in the study. All open injuries, patients who refused to participate in this program, patients who have associated injuries (such as head injury, abdominal injury, or other bone injuries), patients with a subtrochanteric femur fracture, and patients with less than six months of follow-up were excluded.

Ours is a tertiary care hospital with a medical college setup. We have three units in the department. Each unit is assigned a specific day for the emergency and outpatient department (OPD). We included all the patients during the study period. The patients were admitted in their respective units as per the day of arrival in the hospital. Hence, selection bias was avoided. After X-ray scans, the patients were explained the management plan and prognosis. The affected limb was kept in ankle skin traction. Preoperative investigations and anesthesia workups were done. Implant size (long or short) and design (PFN/ PFNA2) were decided at random without any bias as per the unit head's decision. A rough judgment of the nail angle was decided as per the position of the center of the femoral head and the tip of the greater trochanter on the normal side. If the tip of the greater trochanter is higher, which means a coxa vara, an angle of 130° was selected. All the surgeries were performed by unit heads (who is a professor or an associate professor).

Operative procedure

**Figure 1:**
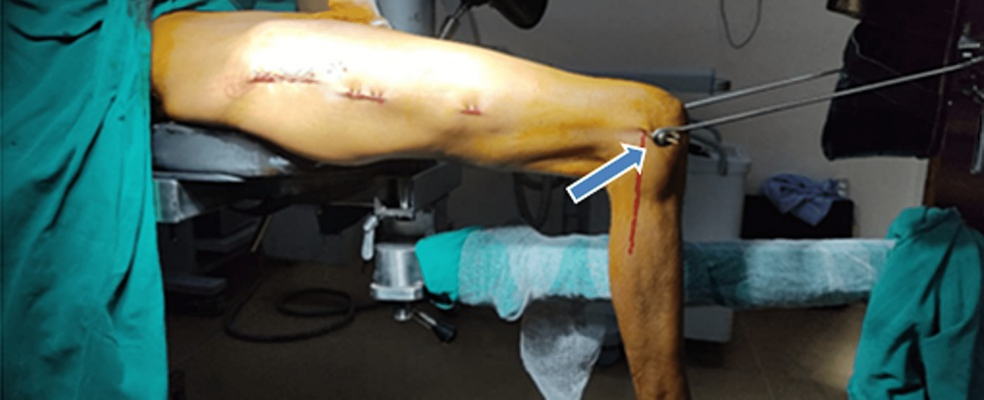
Steinmann pin traction in the distal femur

All standard aseptic and antiseptic precautions were taken. The patients were operated on an orthopedic fracture table in the supine position. In all the cases, distal femur Steinmann pin (ST) skeletal traction with Bohler's stir-up was used (Figure 1).

Implants were from Nebula Surgical Pvt. Ltd. All long nails (PFN/PFNA2) were side-specific. All nails were available in 9, 10, and 11 mm diameters. Long nails were available in lengths from 340 to 420 mm. Short nails were available in lengths of 180 and 250 mm. Helical blades were available in 70 mm to 120 mm lengths.

**Figure 2:**
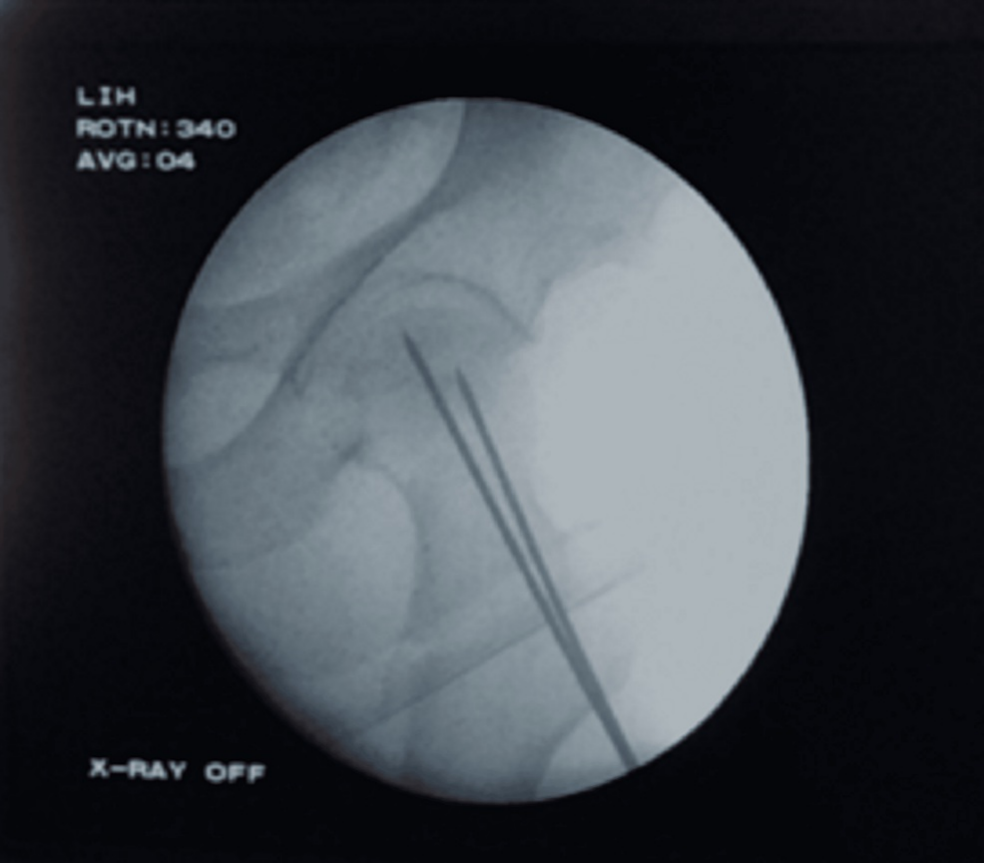
Reduction checked under the C-arm

The reduction was held by inserting ST pin/guide wires in the anterosuperior quadrant of the head and neck, keeping in mind the trajectory of future nail. Adduction was done (which now occurs at the hip joint rather than at the fracture site) to make the greater trochanter more prominent and palpable (in obese patients) for the entry of the nail and checked under the C-arm, as shown in Figure 2.

Entry was made with an awl at the medial slopping edge of the greater trochanter (medial to the tip of the greater trochanter). Entry was confirmed in two views in the C-arm. A cannulated instrument (fossa finder) was inserted and negotiated up to the level of a lesser trochanter. A 2.8 mm guide wire was inserted into the femoral shaft along with the help of a fossa finder. Reaming of the femur was done with the reamer provided with the set in the sizes 8-9-10-11-12. The nail diameter was confirmed preoperatively by the measurement on a true-size radiograph and intraoperatively by a template (under the C-arm) as per the standard intramedullary nail technique. Proximal reaming with a proximal reamer of size 15 mm was done before the insertion of the nail with a sleeve over the lateral cortex to avoid perforation/fracture of the lateral cortex. A proper-size nail was inserted with the zig in a routine manner.

For neck screws, first, the distal guide wire was inserted (parallel to the lower border of the neck) followed by the proximal one. In the case of PFNA2 likewise, a guide wire was inserted through the center of the femoral head up to the subchondral bone.

First, the 6.4 mm derotation screw (anti-rotation) was inserted followed by the 8 mm hip screw (keeping in mind the concept of the tip apex distance). The screws were finally tightened after releasing the traction. For PFNA2, a helical blade of proper size was impacted in an unlocked state, and compression of 5 mm was achieved after final positioning and releasing the traction. This principle helps to prevent the Z-effect or reverse Z-effect (if we do not release the traction before tightening the screws, on transfer from the fracture table, the screw will back out).

**Figure 3:**
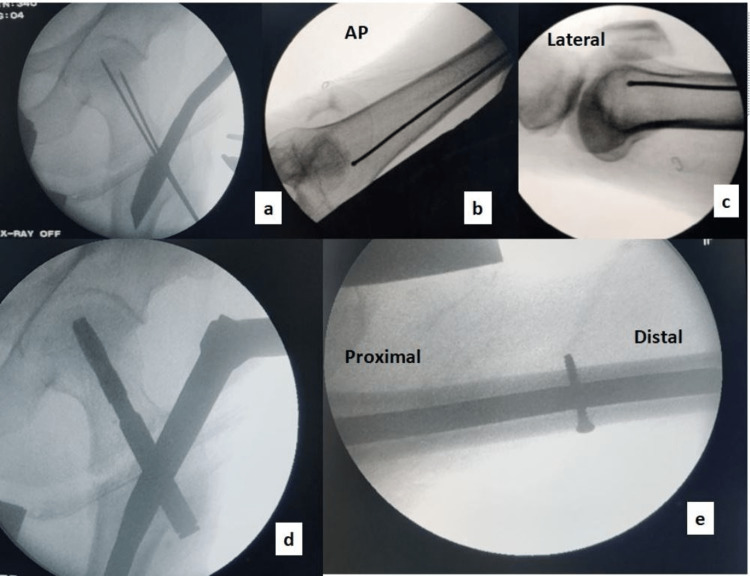
Steps of surgery (image intensifier television (IITV) screen images) A: entry point, B: guide wire in the anteroposterior view, C: guide wire in the lateral view, D: nail and blade fixation, E: distal dynamic locking

Distal locking was done as per the surgeon's choice (both static and dynamic versus only distal dynamic) with 4.9 mm locking screws (Figure 3). Usually, when the surgeon wants some micromotion on mobilization, only the distal dynamic option is selected.

Post-operative X-rays were taken for record purposes and to compare with the X-rays on further follow-ups. Weight-bearing was allowed as per surgeon’s discretion (depending upon the table stability of the fracture) and as per radiological assessment at follow-up. The dressing was checked by the operating surgeon on the third or fourth postoperative day. Intravenous (IV) antibiotics were given for 24-72 hours (usually for 24 hours but extended in patients with diabetes) and intramuscular (IV/IM)/oral analgesics were given till discharge. Routine physiotherapy was given to strengthen the quadriceps muscle. The patient was discharged from the hospital once the wound condition was satisfactory and no clinical signs of infection were noted. Routine follow-up with radiographs was obtained at two, four, six, 12, and 24 weeks until a solid continuous callus formation was observed. Surgical and functional outcomes were assessed during the follow-up visits using the modified Harris hip score (HHS).

## Results

Of the 30 patients operated on for IT femur fracture, we found that the maximum number of patients were in the age group of 51-70 years in both PFN and PFNA2, as shown in Table 1. The overall mean age was 62.84 years; 59 and 66.66 years for PFN and PFNA2, respectively. Age groups were defined as young age (18-30 years), middle age (31-50 years), elderly (51-70 years), and old age (>70 years) groups.

**Table 1:** Age distribution p-value = 0.0256 PFN: proximal femoral nail; PFNA2: proximal femoral nail anti-rotation

Age group (years)	Number of patients			
	Short PFN	Long PFN	Short PFNA2	Long PFNA2
18-30	0	2	0	0
31-50	3	0	2	0
51-70	2	5	6	3
>70	2	1	3	1
Total	7	8	11	4

We observed male preponderance, and among them, the majority were operated with PFNA2, whereas females were operated with PFN, which was purely randomized and without any bias, as shown in Table 2.

**Table 2:** Gender distribution p-value = 0.1394 PFN: proximal femoral nail; PFNA2: proximal femoral nail anti-rotation

Gender	Number of patients	
	PFN	PFNA2
Male	6	11
Female	9	4
Total	15	15

The majority of the patients were operated on with short nails (PFN and PFNA2), as shown in Table 3. All the patients were operated on with 130° nails. Most of the patients were operated on with 10 mm nail diameter (17/30 patients), 380 mm length (long-nail group) (five/11 patients), and 250 mm length (short-nail group).

**Table 3:** Type of implant used p-value = 0.4997 PFN: proximal femoral nail; PFNA2: proximal femoral nail anti-rotation

Type of implant	Short nail	Long nail
PFN	8	7
PFNA2	11	4

With the use of PFNA2, the overall duration of hospital stay was less. Only one patient of the PFN group required a longer hospital stay due to associated comorbidities, as shown in Table 4.

**Table 4:** Duration of hospital stay p-value = 0.0256 PFN: proximal femoral nail; PFNA2: proximal femoral nail anti-rotation

Days	PFN	PFNA2
1-4	1	5
5-10	13	10
>10 days	1	0
Total	15	15

The majority of the patients had associated comorbidity of hypertension (HTN), whereas only a few patients had diabetes mellitus (DM) alone or a combination of both of them, as shown in Table 5. One patient had chronic obstructive pulmonary disease (COPD).

**Table 5:** Comorbidities HTN: hypertension; DM: diabetes mellitus; COPD: chronic obstructive pulmonary disease

Co-morbidity	No. of patients
HTN	12
DM	2
HTN+DM	2
Others (COPD)	1
Total	17

The majority of the patients suffered from fractures due to falls or slippage on level ground (trivial trauma), as shown in Table 6.

**Table 6:** Mode of trauma

Mode	Number of patients
Road traffic accident	9
Slippage/domestic fall	21
Total	30

The overall operative time (incision to wound closure) with the use of short PFNA2 was lesser than that with the use of other designs due to the use of the zig for distal screws, as shown in Table 7. The overall operative time was 77.65 minutes. In the case of PFN, it was 93 minutes, with 89.2 minutes for short PFN and 105 minutes for long PFN. In the case of PFNA2, it was 74 minutes, with 77 minutes for short PFNA2 and 85 minutes for long PFNA2.

**Table 7:** Operative time p-value = 0.6664 PFN: proximal femoral nail; PFNA2: proximal femoral nail anti-rotation

Minutes (mins.)	Short PFN	Long PFN	Short PFNA2	Long PFNA2
60 mins	1	0	4	0
60-75 mins	2	0	6	1
75-90 mins	2	5	1	3
90-120 mins	2	2	0	0
>120 mins	0	1	0	0
Total	7	8	11	4

The use of static versus dynamic locking is shown in Table 8.

**Table 8:** Static vs. dynamic locking p-value = 0.0256 PFN: proximal femoral nail; PFNA2: proximal femoral nail anti-rotation

	Static	Dynamic	Both
PFN	1	8	6
PFNA2	3	9	3

**Figure 4:**
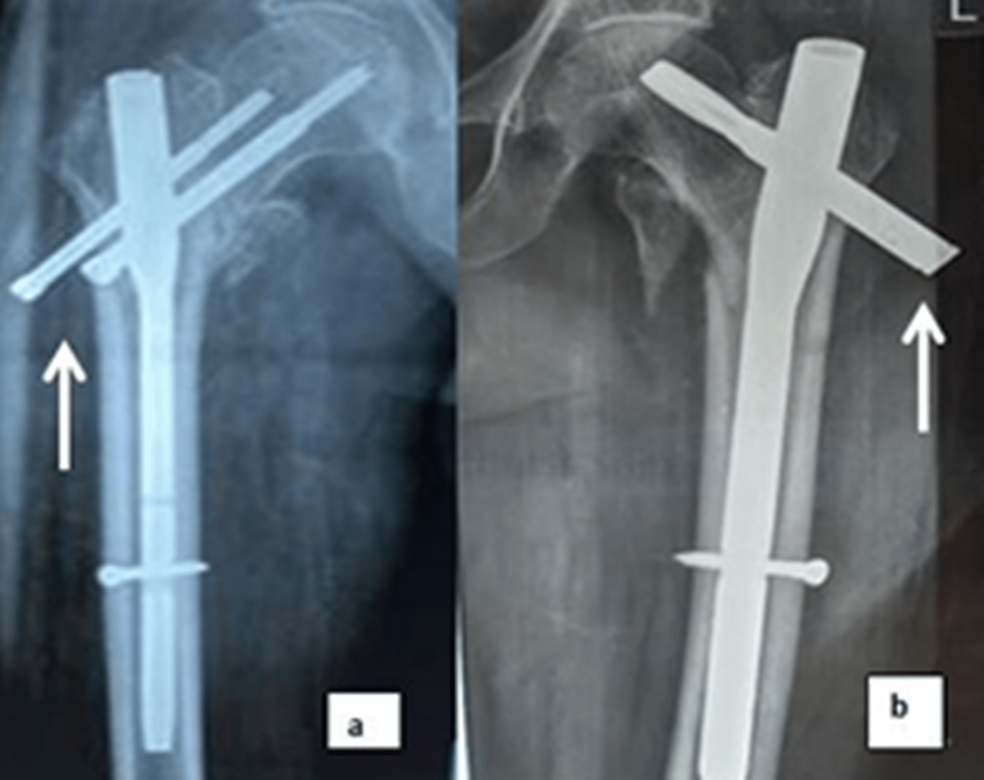
Complications A: reverse Z-effect, B: blade back-out

Few of the patients had the below-mentioned post-operative complications found during subsequent follow-ups, as shown in Table 9 (Figure 4). Deep vein thrombosis (DVT) and fat embolism were noted in the first week. The reverse Z-effect, screw back-out, and superior screw cut-out were noted in the first eight weeks. 

**Table 9:** Complications

Type of complication	No. of cases
Reverse Z-effect/screw back-out	2
Superior screw cut-out	1
Deep vein thrombosis	1
Fat embolism	1
Total	5

Not maintaining the temporary anchorage device (TAD) was the reason for the reverse Z-effect in two cases. However, it cannot be added to the demerits of the implant. In one case, the distal dynamic screw could not be passed (due to the zig mismatch and inability to lock with a free hand despite repeated attempts), so only the static distal locking screw was fixed, which may be a cause for a superior screw cut-out. DVT can be due to a delay in surgery due to comorbidities in one case. The reason for the fat embolism could not be found in one case, but the patient survived.

The union time was nearly the same in the majority of the patients, with an early union seen with the use of PFNA2 nails, as shown in Table 10. The mean union time was 14.2 weeks. In the case of PFN, it was 14.8 weeks, with 13 weeks for short PFN and 16.5 weeks for long PFN. In the case of PFNA2, it was 13.6 weeks, with 14 weeks for short PFNA2 and 12.5 weeks for long PFNA2.

**Table 10:** Union time p-value = 0.0256 PFN: proximal femoral nail; PFNA2: proximal femoral nail anti-rotation

Union time (in weeks)	Short PFN	Long PFN	Short PFNA2	Long PFNA2	Total (%)
Up to 10 weeks	2	0	3	2	7 (23.33%)
10-16 weeks	4	6	7	2	19 (63.33%)
>16 weeks	1	2	1	0	4 (13.33%)
Total	7	8	11	4	30

As per Boyd and Griffin's classification, the majority of our patients had type II fractures, as shown in Table 11.

**Table 11:** Fracture pattern as per Boyd and Griffin's classification p-value = 0.4625 PFN: proximal femoral nail; PFNA2: proximal femoral nail anti-rotation

Type	Number of patients				Total (%)
	Short PFN	Long PFN	Short PFNA2	Long PFNA2	
I	2	1	3	2	8 (26.66%)
II	3	1	8	1	13 (43.33%)
III	1	3	0	0	4 (13.33%)
IV	1	3	0	1	5 (16.66%)
Total	7	8	11	4	30

The majority had a union time between 10 and 16 weeks with an average of 14.2 weeks. Most of them were operated with short PFNA2. The overall modified HHS at the final follow-up was nearly the same with slightly better results with the use of PFNA2, as shown in Table 12 (p-value = 0.0077, signifying that PFNA2 is statistically better than conventional PFN).

**Table 12:** Modified Harris hip score at the final follow-up p-value=0.0077 (comparing PFN vs. PFNA2) PFN: proximal femoral nail; PFNA2: proximal femoral nail anti-rotation; HHS: Harris hip score

Implant	Average modified HHS	
	Short	Long
PFN	82.38	83.5
PFNA2	84.7	83.4

**Figure 5:**
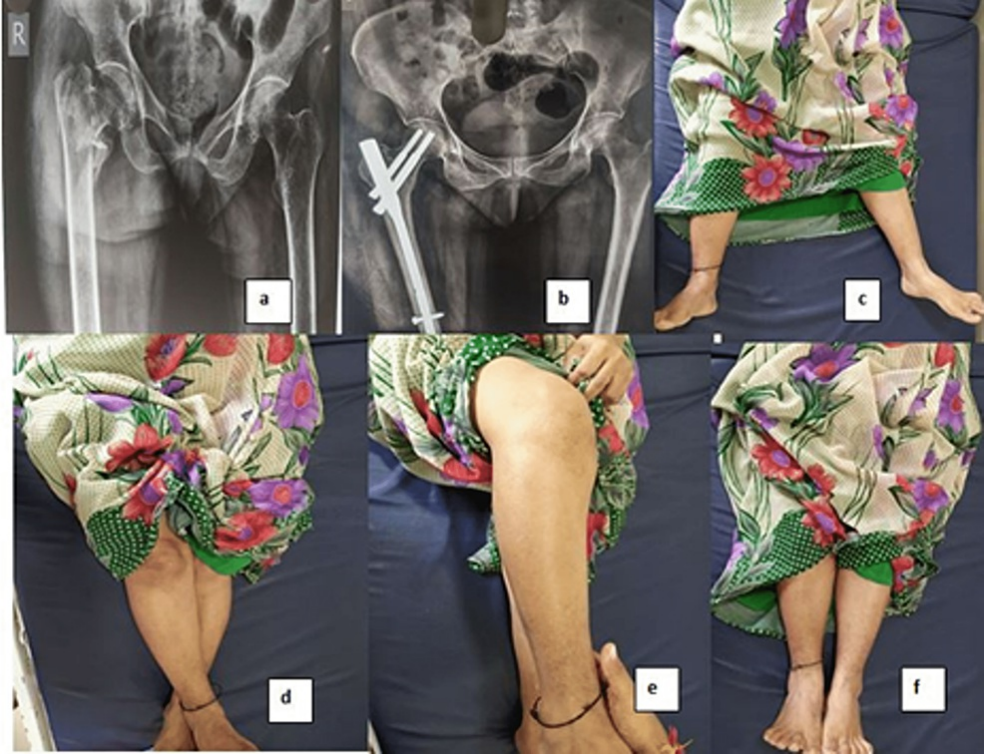
Images of patients showing excellent results A: trauma radiograph, B: union radiograph, C: abduction, D: adduction, E: flexion, F: no limb length discrepancy (pelvis squared)

We also found good results when using short PFNA2 as compared to other nail designs, as shown in Table 13. We had excellent results in seven patients and good results in 14 patients (Figure 5).

**Table 13:** Interpretation of the modified Harris hip score at the final follow-up PFN: proximal femoral nail; PFNA2: proximal femoral nail anti-rotation

Interpretation	Number of patients				Total (%)
	Short PFN	Long PFN	Short PFNA2	Long PFNA2	
Excellent	1	2	3	1	7 (23.33%)
Good	3	3	6	2	14 (46.66%)
Fair	3	3	2	1	9 (30%)
Total	7	8	11	4	30

All the patients were operated with closed reduction except one (due to soft tissue interposition). The average blood loss was 100-120 ml when the patient was operated on with short PFN/PFNA2, and it was 180-200 ml when the patient was operated on with long PFN/PFNA2. Blood loss was measured as per weight and the area of the mop soaked during the surgery. The drain amount was calculated too.

The average fluoroscopy time in the short PFN/PFNA2 was 80-100 seconds, which was lower compared to the long PFN/PFNA2 with an average of 140-160 seconds. None of the patients operated on with short PFN/PFNA2 experienced anterior thigh pain. All the patients were satisfied and returned to their preoperative job. None of them needed reoperation for the same injury.

## Discussion

Operative treatment in the form of intramedullary fixation permits early rehabilitation and functional recovery, and hence it has become the treatment of choice for most cases of IT fracture. Among the various types of nails available, PFN is the most commonly used, but recently the use of PFN with a helical anti-rotation blade (PFNA2) has gained popularity. Both nail designs are available in short and long options. There are no scientific studies that show clinical and functional outcomes comparing all four options. Thus, we studied clinical and functional outcomes comparing both nail designs (PFN and PFNA2) and both sizes (short and long). We prospectively studied all four variants of the nails avoiding selection bias.

Li et al. [9] in their study on a clinical evaluation of PFNA2 for the treatment of IT fractures showed that it had the advantages of a simple operation, few complications, and good clinical efficacy for the treatment of IT fractures. However, the evaluation of its long-term efficacy and risk of other complications requires a large-sample, multi-centric observational study.

Kashid et al. [10] in their comparative study on the management of unstable trochanteric fractures showed that PFNA significantly reduces the operative time, amount of blood loss, and fluoroscopic imaging time as compared to PFN. However, PFNA had no significant benefits over PFN in terms of post-operative functional recovery or complications.

Gururagavendra et al. [11] in their study of the management of unstable IT fracture concluded that the mean time for radiological union and functional outcome was comparable in both groups. The mean operative time and blood loss were lesser in the case of PFNA2. Their study concluded that both implants have comparable radiological and functional outcomes for unstable IT fracture except for less surgical time and blood loss in PFNA2. Complications, such as proximal migration of the spiral blade into the hip joint and lateral thigh pain, were not seen in this study.

Singh and Bhartiya [12] in their study showed equally good functional outcomes following the fixation of unstable trochanteric fractures. PFNA offered no significant benefits over PFN in terms of postoperative complications.

Gardenbroek et al. [13] in their study on PFNA found that the union with PFNA does not improve the position of the implant in the femora­­l head compared with PFN. However, the risk of secondary complications and the need for reoperation is significantly higher in patients who were treated with PFN.

Our study was comparable to the above-mentioned studies in terms of demographic details showing male preponderance and the occurrence of the fracture in the elderly age group. Other studies had also taken an equal number of cases of each of the nail types as in our study. Trivial trauma was the most common mode of occurrence. The type of implant chosen was random without any confounding factors or biases comparable to other studies.

Our study matches with previous studies in terms of lesser operative time, lesser complications, and lesser hospitalization using PFNA2 nail design. There was no major difference in terms of intraoperative blood loss as compared to previous studies. In our study, the average fluoroscopy time was greater compared to other studies. The average number and type of complications in our study were similar to most other studies. Ours is a tertiary care setup, so we got patients from other states. Such patients preferred to get discharged after suture removal to avoid to-and-fro expenses for dressing. This may be one of the reasons for longer hospital stays. The use of a distal dynamic locking screw in the majority of the patients can get better compression at the fracture site once the patient starts weight bearing and decrease the chances of the Z-effect, reverse Z-effect, screw back-out, and screw cut-out.

There was no major difference in the average follow-up period as compared to other studies. The mode of classification of fracture pattern and the union time matched most of the studies. No one from our study required reoperation due to post-operative complications as was the case in some of the previous studies. Our study’s interpretation using the modified HHS matched previous studies, and we had significant similarities in terms of the percentage of patients with excellent and good results.

The randomized selection of implant design and prospective nature are strengths of our study. Randomized patient selection irrespective of fracture type avoids confounders. Our study was unique, as very less studies are reported in the literature, which compared short and long nails in PFN and PFNA2. It was also unique in terms of comparing outcomes and advantages of only distal dynamic screws over static locking or the use of both static and dynamic screws.

A relatively small sample size, shorter follow-up period, and single-center study can be considered our limitations. A larger sample size with a longer follow-up with a multicentric study would have made a better assessment of the long-term outcomes of such a problem.

## Conclusions

PFNA2 is the implant of choice in elderly patients with osteoporotic bone. It has less operative time, which is required in such patients with medical comorbidities; hence, it has marginal superiority over PFN. Short-nail design results in less operative time and less blood loss. Complications are relatively less with PFNA2. Following all surgical steps systematically avoids complications in both groups.

Only distal dynamic locking allows fracture collapse, better compliance, and early union of the fracture. There is no difference in the union time between short and long nail designs. Clinical and functional outcomes do not change at the final follow-up with the use of PFN or PFNA2.
